# The Elizabeth Garrett-Anderson Hospital

**Published:** 1924-04

**Authors:** 


### THE ELIZABETH GARRETT-ANDERSON HOSPITAL.

The fifty-second annual meeting of the Elizabeth Garrett Anderson Hospital, Euston Road, corresponded with "Daffodil Day," which no doubt accounted for the profusion of those cheerful and effective blooms that decorated every ward table in the hospital on March 14 and brightened the large entrance hall, where tea lay in readiness for guests attending the meeting. The chair was taken by Lord Riddell, and among those present were Mrs. Fawcett, LL.D., sister of the late Dr. Garrett-Anderson, founder of the hospital, Miss Aldrich-Blake, M.D., M.S., Dr. Jane Turnbull, Mr. Gordon Pollock, Chairman of the Managing Committee, and many others. The hospital, which was the first to be staffed by women doctors, began in quite a small way with very few beds, and in spite of much opposition, in 1866, when medical women were comparatively unknown. But the spirit of adventure and courage which upheld its founder through all her many difficulties, descended upon those coming after her, and the institution has never lacked these energising attributes, nor has it ever wanted for friends.

### Moving Forward.

Addressing the meeting, Lord Riddell stated with great satisfaction that the freehold of the site on which the hospital and its annexes stood had now been purchased as the result of a bequest, amounting to nearly £10,000, under the will of the late Mrs. Lorillard, a New York lady, the institution having been selected by her executor as the "most deserving charity" for the legacy. While, therefore, the hospital owes its origin to the devotion of one woman, its work has been consolidated by another. Because of the increasing demand for beds the existing accommodation of 75 beds at Euston Road and 28 at the High Barnet Country Branch, is proving quite insufficient, and an appeal is being made for contributions towards the £50,000 required for extensions. Plans for the new buildings have been approved by the King's Fund, and the work will be begun as soon as £25,000 is in hand.

### The Value of an Almoner.

Another item of interest mentioned at the meeting was that the new appointment made in 1923 of an Almoner, Miss Ronaldson, has proved a most successful advance in administration.

